# Configuration-Specific Insight into Single-Molecule Conductance and Noise Data Revealed by the Principal Component Projection Method

**DOI:** 10.1021/acs.jpclett.3c00677

**Published:** 2023-05-30

**Authors:** Z. Balogh, G. Mezei, N. Tenk, A. Magyarkuti, A. Halbritter

**Affiliations:** † Department of Physics, Institute of Physics, Budapest University of Technology and Economics, Műegyetem rkp. 3, H-1111 Budapest, Hungary; ‡ ELKH-BME Condensed Matter Research Group, Műegyetem rkp. 3., H-1111 Budapest, Hungary

## Abstract

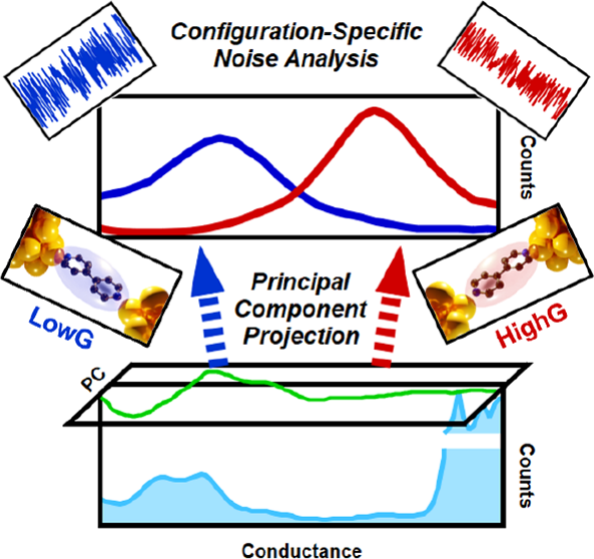

We explore the merits of neural network boosted, principal-component-projection-based, unsupervised data classification in single-molecule break junction measurements, demonstrating that this method identifies highly relevant trace classes according to the well-defined and well-visualized internal correlations of the data set. To this end, we investigate single-molecule structures exhibiting double molecular configurations, exploring the role of the leading principal components in the identification of alternative junction evolution trajectories. We show how the proper principal component projections can be applied to separately analyze the high- or low-conductance molecular configurations, which we exploit in 1/f-type noise measurements on bipyridine molecules. This approach untangles the unclear noise evolution of the entire data set, identifying the coupling of the aromatic ring to the electrodes through the π orbitals in two distinct conductance regions, and its subsequent uncoupling as these configurations are stretched.

The field of single-molecule electronics (SME) has developed a broad range of experimental methods to gain insight into the properties of the ultimate smallest conductors, where a single molecule bridges two metallic electrodes.^1−5^ The creation of single-molecule nanowires heavily relies on the so-called break junction methods, where the fine mechanical rupture of a metallic nanowire is utilized first to establish a single-atom metallic nanojunction and afterward to contact a single molecule between the two apexes of a broken atomic junction.^1,2,4,6,7^ The basic output of the break junction measurements is a statistical ensemble of conductance vs electrode separation traces, from which the fingerprints of the single-molecule configurations can be visualized on conductance histograms. This analysis is frequently extended by more delicate experimental methods, like force,^2,8−11^ noise,^11−22^ thermoelectric power,^2,23,24^ thermal conductance,^25−27^ quantum conductance fluctuation,^28,29^ or superconducting subgap spectroscopy measurements.^30,31^ The combination of such methods was found to be extremely efficient in resolving the fine details of the structural and electronic properties of single-molecule nanowires.

In spite of this broad range of diagnostic tools, single-molecule electronics still suffers from the diversity of the data, i.e., the formation of a broad range of various single-molecule configurations during the repeated opening and closing of the junction. Conductance histograms are sufficient to resolve the *conductance of the most probable molecular configurations*. However, if a deeper insight is required, or further properties are also analyzed in parallel with the conductance data, it is useful to filter out the traces indeed reflecting the molecular configuration under focus; otherwise, the mixture of alternative configurations in the data set may obscure the result of the analysis. This kind of data filtering is a traditional ingredient of break junction data analysis mostly relying on well-defined, but custom-created filtering algorithms.^32−35^ The recent progress of machine learning (ML) based data analysis has brought a novel perspective to the field of SME as well.^36−45^ However, in this field the widespread supervised ML approaches are less favored, as prelabeled example traces are usually not available, and the manual selection of training traces is also unreliable and unwanted. Instead, the *unsupervised* identification of the relevant trace classes is desired, which is indeed possible by proper ML approaches, including reference-free clustering methods, or autoencoder-based clustering.^37,38,40−42,46^

Here we examine an alternative unsupervised feature recognition approach relying on the *internal correlations* of the data set. This method utilizes the cross-correlation analysis of the conductance traces.^33,45,47−50^ The leading principal components (PCs), i.e., the most important eigenvectors of the 2D correlation matrix, identify the most relevant correlations in the data set offering a unique tool for the efficient identification of the relevant trace classes. The principal component projection (PCP) method was first introduced and successfully applied to identify artificially mixed traces of different molecules.^51^ Later, the authors of this paper demonstrated the sorting of molecular and tunneling traces by the PCP method.^52^ In the latter work, it was also shown that the classification accuracy is significantly improved if the PCPs are only used to automatically generate the most characteristic traces for the two trace classes, which are then used as the training data set of a simple neural network (NN).^52^

Inspired by the initial success of the PCP approach, here we perform a thorough analysis on the benefits, constraints, and general anatomy of this method via the investigation of room-temperature Au–4,4′-bipyridine (BPY)–Au^7,45,52−58^ and Au–2,7-diaminofluorene (DAF)–Au^11^ molecular nanowires. Both systems exhibit two different molecular configurations; i.e., alternative junction evolution trajectories are conceivable. We demonstrate that the PCP-based recognition of highly relevant, alternative trace classes is not incidental; rather, *all* the leading principal components provide highly relevant classification criteria highlighting features, which are mixed in the entire data set. Furthermore, we demonstrate the merits of this efficient and robust data filtering tool in more complex single-molecule measurements, where a configuration-specific insight into the data is desired. The latter is demonstrated by our PCP-aided, configuration-specific 1/f-type noise measurements on BPY single-molecule junctions. These measurements investigate the scaling of 1/f-type noise amplitude with the junction conductance, which is a specific device fingerprint distinguishing through-bond and through-space molecular coupling.^11,12,14,18,21^ In the latter case, a weak bond is involved in the transport, where tiny distance fluctuations exponentially convert to a significant conductance noise. This yields conductance independent relative conductance noise, Δ*G*/*G* similar to a tunnel junction, where the exponential dependence of the conductance on the distance, *G* ≈ exp(−β·*d*) converts to Δ*G*/*G* ≈ const. for a constant Δ*d* distance fluctuation.^12,14^ As a sharp contrast, in the case of through-bond coupling a strong chemical bond fixes the molecule–apex distance, and therefore the noise is not related to distance fluctuations in the bond, but rather to close-by atomic fluctuations of the metallic apex. This phenomenon results in a definite increase of the relative conductance noise with decreasing junction conductance yielding a scaling exponent close to Δ*G*/*G* ≈ *G*^–0.5^.^12,14^ Our noise analysis on BPY molecules yields a complex, unclear Δ*G*/*G* vs *G* dependency. The PCP-based configuration-specific noise analysis, however, fully clarifies the noise characteristics of the two distinct molecular configurations, providing a refined insight into the bond evolution.

*Basics of the Measurements*. The break junction measurements are performed by scanning tunneling microscopy (STM) or mechanically controllable break junction (MCBJ) methods, where the mechanically sharpened Au tip of a custom-designed STM setup is brought into contact with a gold thin film sample covered by the target molecules, or a gold wire is broken in a molecular environment using a three-point bending geometry.^1^ In both cases, the rupture and the closing of the junction are repeated several thousand times, characterizing each rupture process by a conductance vs electrode separation (*G*(*z*)) trace. From each measured trace, indexed with *r*, a single-trace histogram denoted by *N*_*i*_(*r*) = *N*(*G*_*i*_, *r*) is calculated by dividing the conductance axis to discrete bins (*G*_*i*_) and counting the number of data points in each bin. The conductance histogram of the entire data set,  is obtained by averaging the single-trace histograms for all traces. These simple conductance histograms can be expanded to two-dimensional conductance histograms,^53,59^ where the displacement information is also visualized. The such-obtained one-dimensional and two-dimensional conductance histograms for room-temperature Au-BPY-Au and Au-DAF-Au single-molecule junctions are presented in Figure 1a,b (BPY) and Figure 1d,e (DAF) (see the blue area graphs in the 1D histograms). In both cases, the formation of single-atom Au junctions is reflected by a sharp peak at the quantum conductance unit, *G*_0_ = 2*e*^2^/*h*, and both molecular systems exhibit double molecular configurations, one with higher and one with lower conductance (HighG/LowG). In the case of BPY, it is suggested that at a smaller electrode separation, the molecule can bind such that both the nitrogen linker and the aromatic ring is electronically coupled to the electrode, yielding a higher (HighG) single-molecule conductance (Figure 1g1). Upon further pulling, the molecule slides to the apex, and only the linkers couple to the electrodes (Figure 1g2) yielding a decreased (LowG) conductance value.^53−55,60^ In the case of DAF the HighG/LowG configurations are interpreted as *monomer*/*dimer* molecular junctions (see Figure 1h1,h2).^11^

**Figure 1:**
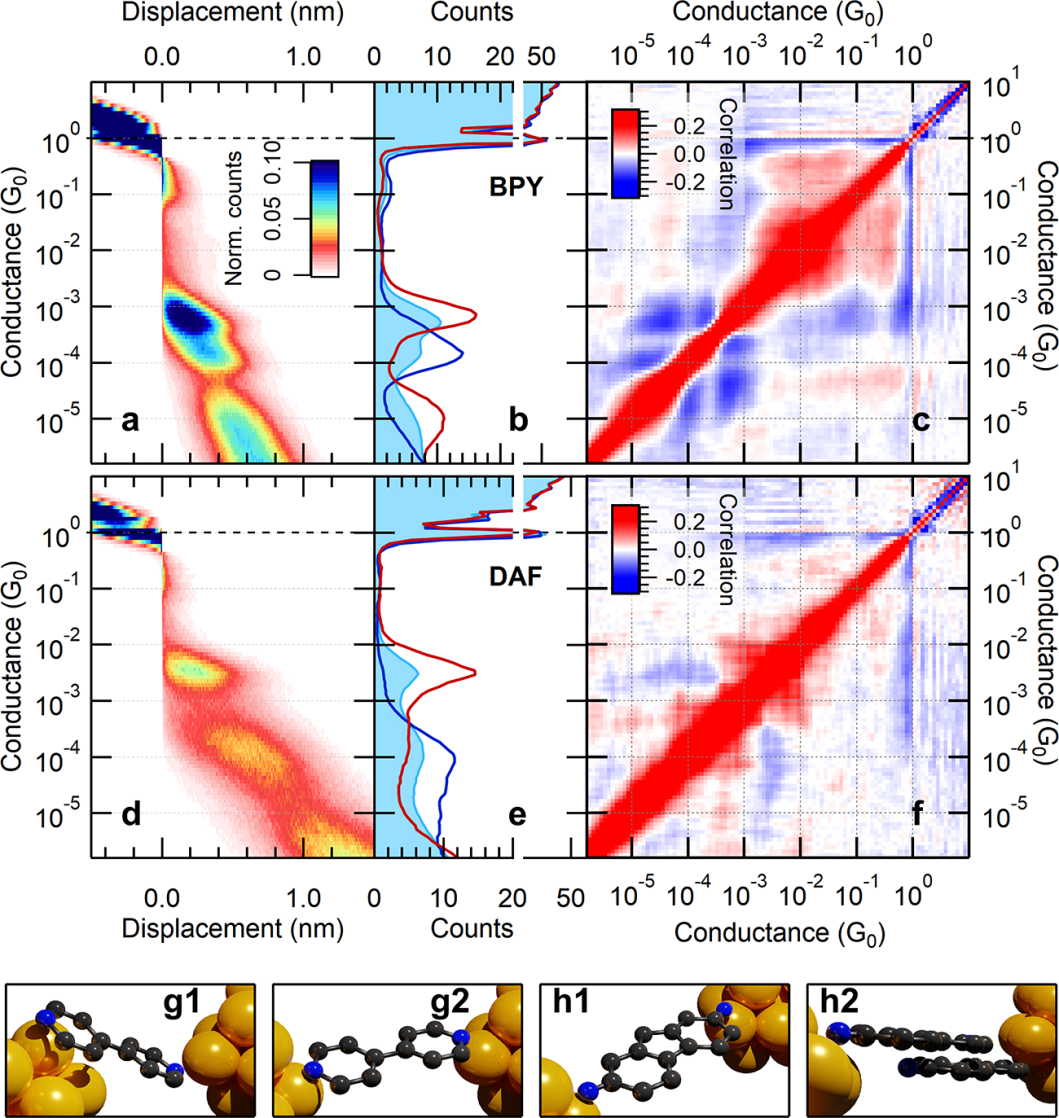
Histograms and correlation plots of BPY and DAF molecules. The top row represents the characteristics of BPY molecules, including the 2D histogram (a), the 1D histogram (blue area graph in panel (b)), and the *C*_*i*,*j*_ correlation plot (c). The red and blue lines in panel (b) represent the histograms of the data subsets obtained by EPCP analysis (see text). The second row similarly demonstrates the 2D histogram (d), the 1D histogram (e), and the correlation plot (f) for DAF molecules. On the 2D histograms the origin of the displacement axis is set to the position where the conductance trace crosses *G*_ref_ = 0.5G_0_. Panels g1 and g2 illustrate the hypothesized HighG and LowG BPY configurations, whereas h1 and h2 illustrate the HighG (monomer) DAF configuration and the LowG (dimer) DAF configuration.

*Basics of Principle-Component-Projection-Based Conductance Trace Classification*. The one- and two-dimensional conductance histograms represent an average behavior of the conductance traces. However, further, highly relevant information is obtained from the correlation matrix of the data set,^47,48^ highlighting the correlated deviations from the average behavior:
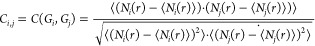
The correlation matrices of the Au-BPY-Au and Au-DAF-Au systems are demonstrated in Figure 1c,f, both exhibiting a rich correlation structure. Some aspects of the correlation matrix can be interpreted with proper experience,^33,45,47−50^ but the complexity of analyzing 2D correlation matrices is significantly reduced by concentrating on the *most important correlations* represented by the *P*_*i*_^(*n*)^ principal components, i.e., the eigenvectors of the correlation matrix.^51^ Here, the *n* label sorts the principal components according to the descending order of the corresponding eigenvalue.

The principal component projection of a certain conductance trace is obtained as PCP^(*n*)^(*r*) = ∑_*i*_*P*_*i*_^(*n*)^·*N*_*i*_(*r*), i.e., the scalar product of the principal component and the single-trace histogram. According to the proposal of J. Hamill and co-workers, the PCP^(*n*)^(*r*) < or > 0 relation can be used to sort the traces to two relevant trace classes.^51^ This idea was successfully applied to separate artificially mixed traces of two different molecular systems. It was also shown that the zero PCP value is not necessarily the proper threshold for the selection, and the accuracy of PCP-based data classification can be further refined by relying only on the traces with the largest positive/negative PCP (e.g., selecting 20% – 20% of all traces).^52^ If the classification of the *entire* data set is not targeted, solely the traces with these extreme principal component projection (EPCP) can be considered as a subset of the traces best representing some distinct behavior. Alternatively, these *characteristic traces* can be applied as the training data set for a simple double-layer feed-forward neural network.^52^ In this combined PCP-NN method the neural network *learns* the important features from these training traces, and then generalizes for traces with less obvious characters. The decision-making process of this simplest possible neural network is well-represented by the so-called summed weight product,^52^ SWP_*i*_ = ∑_*j*_*W*_*i*,*j*_^(1)^·*W*_*j*_^(2)^, where *W*_*i*,*j*_^(1)^/*W*_*j*_^(2)^ is the neural weight matrix/vector between the input layer and the hidden layer/hidden layer and output neuron of the NN, respectively. If SWP_*i*_ is a large positive/negative number for a certain input, a large input value (i.e., a large histogram count) pushes the decision toward one or the other selection group.

*Principle Component Projection Analysis of Room-Temperature Bipyridine and Diaminofluorene Single-Molecule Junctions*. In our previous work the PCP-NN method was successfully applied in low-temperature single-molecule measurements,^52^ where the molecular pick-up rate was relatively low, and therefore, it was a primary task to separate molecular and nonmolecular (tunneling) traces, which was achieved by the projection to the second principal component. In the following, we further demonstrate the merits and constraints of the EPCP and the combined PCP-NN method by analyzing the trace classes according to *all the leading principal components* in room-temperature measurements with BPY and DAF molecules. These measurements exhibit ≈100% molecular pick-up rate, and therefore, instead of molecular/tunneling distinction, we rather focus on the analysis of the possible relations of the LowG and HighG molecular configurations. Prior to this detailed analysis, however, we first illustrate the results of the EPCP analysis for elected principal components (Figure 1b,e). The red/blue curves show the conductance histograms for the traces with the largest positive/negative principal component projection, relying on *P*^(3)^ (*P*^(2)^) for BPY (DAF) molecules. All selections include 20% of all traces, and for both molecules, one selection exhibits the dominance of the HighG configuration and the suppression of the LowG configuration, and the other selection behaves vice versa. The possibility of this separation is already surprising, as the 2D histograms of both molecular systems (Figure 1a,d) suggest that first the HighG configuration appears, and the LowG configuration only forms upon further stretching. This scenario would imply that the LowG configuration appears together with the HighG one. As a very sharp contrast, the EPCP method demonstrates that one can find a significant portion (in this case 20%) of the traces, where the pronounced LowG peak can be observed without any significant HighG weight. This means that the *average behavior* represented by the 1D and 2D histograms may obscure possible highly relevant junction evolution trajectories, which can be, however, uncovered by the proper principal component projection.

Further on, we extend the EPCP method by the neural network supplement and analyze the obtained trace classes for the four leading principal components in room-temperature Au-BPY-Au junctions (Figure 2). The rows of the figure respectively represent the analysis according to the first, second, third, and fourth principal components. For a certain principal component, the separated two trace classes are demonstrated both by 2D conductance histograms (Figures 2a1–a4 and b1–b4), and by 1D conductance histograms (Figure 2c1–c4). In the latter case, the light blue background histogram represents the reference histogram for the whole data set, whereas the red and blue lines demonstrate the histograms of the two data sets obtained by the PCP-NN analysis. In the fourth column (Figure 2d1–d4) the actual principal component (dark green lines) and the summed-weight products of the neural network (light green lines) are demonstrated. Naturally, the selected one/other trace class exhibits pronounced weights in those conductance regions, where the PC and the SWP exhibit large positive/negative values.

**Figure 2:**
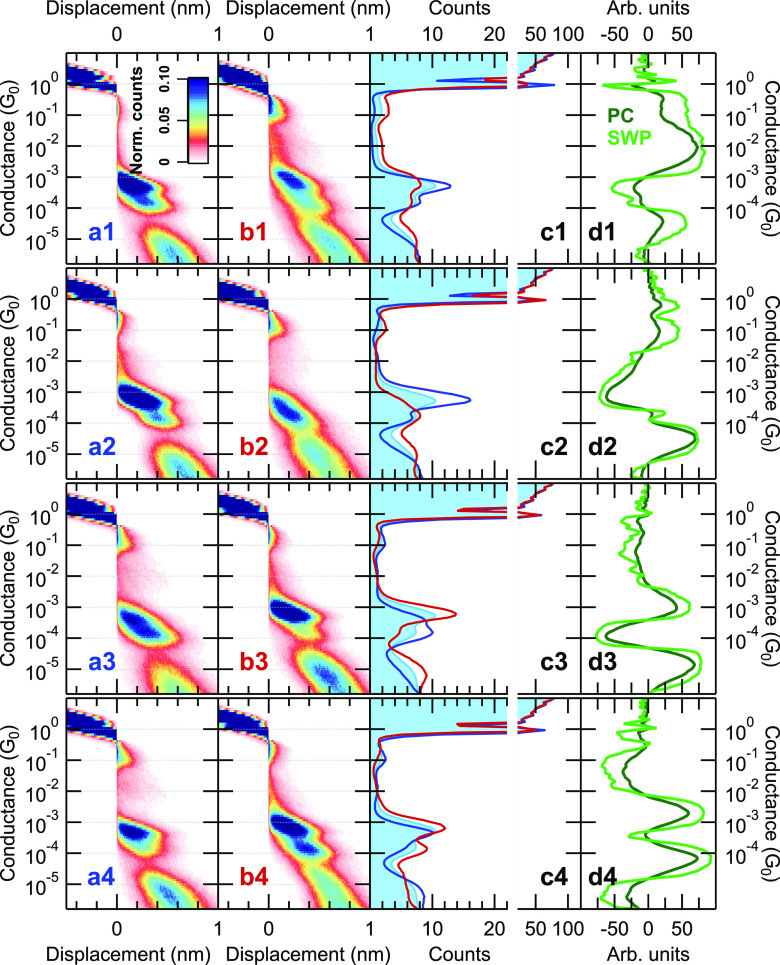
PCP-NN analysis of Au-BPY-Au single-molecule junctions according to the four leading principal components. The analysis according to *P*^(*n*)^ is presented in the *n*th row; the corresponding principal components are shown by dark green lines in panels (d1–d4). The PCP-NN method classifies all the traces to two groups, trace class (a) and (b). The corresponding 2D histograms are respectively shown in panels (a1–a4) and (b1–b4), whereas the 1D histograms are shown in panels (c1–c4) by blue lines (trace class (a)) and by red lines (trace class (b)). As a reference, the blue area graphs represent the 1D histogram for the whole data set. In panels (d1–d4), the light green lines represent the corresponding summed-weight-product of the neural network. The entire data set includes 18301 traces, from which all the traces are classified into one or the other trace class. For the various principal components, the number of traces in a trace class varies in the range of 48.5–51.5% of all traces. For these PCs, the eigenvalues normalized to the eigenvalue of *P*^(1)^ are respectively 1; 0.853; 0.746; 0.567.

First of all, it is clear from Figure 2d1–d4 that the SWPs very much resemble the principal components for all the PCs. In the case of panels d2 and d3, the shape of the PC and the SWP is almost identical, whereas, in case of panels d1 and d4, the SWP refines and enhances the features of the PC; i.e., significantly sharper transitions are observed between the negative and positive regions. This already underpins the added value of the neural network extension of the simple PCP analysis: whereas the NN analysis basically learns the internal correlations of the data set from the PCP, it is able to define more precise and sharper boundaries between the relevant conductance regions. Furthermore, we emphasize again that in the case of the simple PCP method, the best threshold PCP value between the two trace classes is ill-defined (it is not generally zero), but the NN extension is able to find the optimal boundary between the two trace classes.

The analysis according to the *first principal component* enhances/suppresses both histogram peaks in the molecular region compared to the entire data set. The background counts outside of the region of the molecular peaks behave vice versa; these are strongly suppressed/enhanced for the trace class (a)/(b) (see the blue and red lines in Figure 2c1, and the corresponding 2D histograms in Figure 2a1/a2, where the blue/red label color matches the linecolor of the subset histograms in panel c1). Interestingly, the weight in the single-atom (1G_0_) region and a bit higher conductance (≈1.2–1.5 G_0_) is also a significant part of the data filtering. For trace class (a)/(b), the 1G_0_ peak is significantly enhanced/suppressed, whereas the weight at somewhat higher conductance is suppressed/enhanced, respectively. This highlights the importance of *precursor* effects in single-molecule measurements: in many cases the single-atom features are able to forecast the subsequent molecular features exhibiting either positive^61^ or negative^33,44,48,62,63^ correlations between the two conductance regions. In panel (c1) the *G* = 1G_0_ configuration exhibits a strong positive correlation with the molecular conductance region (see Figure S3 in the SI for the magnified, linear-scaled single-atom region of the histograms in panel (c1)) which might arise from two different physical phenomena. One can consider a *contamination model*, where a contaminating molecule spoils the formation of the clear 1G_0_ single-atom configuration, and rather a broader weight is observed at a somewhat larger conductance (similar to our prior Ag-CO-Ag single-molecule study^33^). In this case, we argue, that well-defined BPY single-molecule junctions are best formed after the rupture of a well-defined single-atom Au junction with 1G_0_ conductance. Any contamination that spoils the formation of this clean single-atom structure also hinders the formation of a well-defined Au-BPY-Au single-molecule structure, but rather enhances the background weight above the single-molecule and single-atom conductance. But alternatively a *stabilization model*(61) can also be considered, where the BPY molecule is bound parallel to the single-atom Au junction and thereby stabilizes it, yielding longer single-atom plateaus and a higher peak in the histogram. Therefore, the PCP-NN analysis according to the first principal component is efficient in separating *ideal* molecular traces with well-defined Au-BPY-Au single-molecule configurations from *obscured* molecular traces. The PCP-NN analysis also highlights the precursors of these trace classes in the 1G_0_ conductance region.

While *P*^(1)^ filters the traces by suppressing or highlighting both molecular peaks, the further principal components perform a peak-specific classification. In case of *P*^(2)^, the HighG molecular peak is either enhanced or suppressed, and the LowG molecular peak is left unchanged (Figure 2a2–c2); *in the case of P*^(3)^, either the HighG peak is enhanced and the LowG peak is suppressed, or vice versa (Figure 2a3–c3); *in the case of P*^(4)^, the LowG peak is enhanced or suppressed, whereas the HighG peak is left more-or-less unchanged (Figure 2a4–c4). Although the PCP-NN analysis does not uncover the physical origin of these various trace classes, it makes it very clear that the *average* and well-known junction evolution seen on the 2D conductance histogram of the BPY molecules (Figure 1a) is definitely not a single *representative* junction evolution. While the former (Figure 1a) implies the sequential formation of the HighG and LowG configurations on the *same trace*, the PCP-NN-based data sorting very clearly highlights the presence of various fundamentally different junction evolution trajectories, which all represent a significant portion of the traces. More importantly, these possible significant junction evolution trajectories are not sorted according to some subjective manual data filtering criterion, nor by a black-box-like high-complexity machine learning method; rather, they rely on the well-defined internal correlations of the data set.

Next, we perform a similar analysis on *Au-DAF-Au single-molecule junctions* (Figure 3). *The analysis according to P*^(1)^ (Figure 3a1–d1) provides a somewhat similar result to the BPY molecules: trace class (a) yields the suppression of both molecular peaks, whereas trace class (b) enhances both molecular peaks. The effect of the *projection to P*^(2)^ (Figure 3a2–d2) is similar to the *P*^(3)^ projection in the case of the BPY molecules; i.e., in trace class (a) the HighG peak is suppressed, and the LowG peak is enhanced, and vice versa in trace class (b). In the case of DAF molecules, the further principal component projections (*P*^(3)^ and *P*^(4)^) show very similar results to the *P*^(2)^ projection.

**Figure 3:**
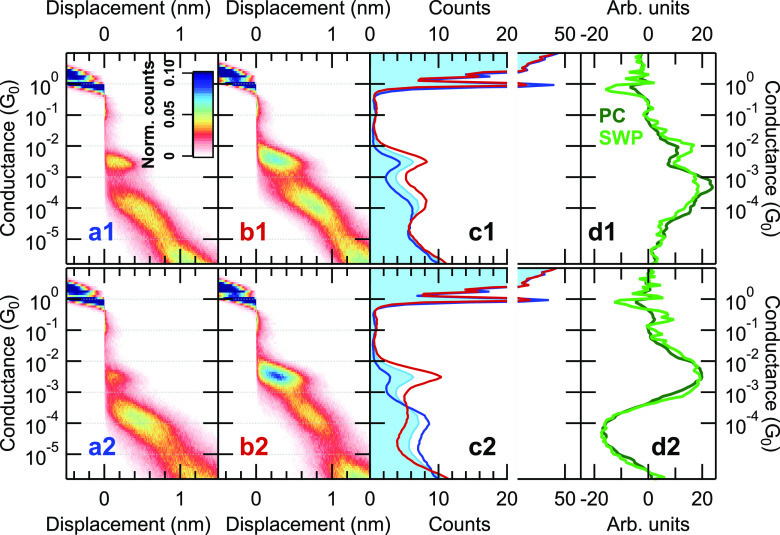
PCP-NN analysis of Au-DAF-Au single-molecule junctions. The analysis according to *P*^(1)^ and *P*^(2)^ is respectively presented in the first and second row; the corresponding principal components are shown by dark green lines in panels (d1, d2). Panels (a1, a2) and (b1, b2) respectively demonstrate the 2D histograms of trace classes (a) and (b). The corresponding 1D histograms are respectively shown by blue and red lines in panels (c1, c2) in comparison to the histogram of the entire data set (light blue area graph). In panels (d1, d2) the light green lines represent the corresponding summed-weight-product of the neural network. The entire data set includes 7001 traces. The selected trace classes include 45–55% of all traces. For these PCs the eigenvalues normalized to the eigenvalue of *P*^(1)^ are respectively 1; 0.889.

To demonstrate the robustness of the PCP-NN analysis, we have also investigated its sensitivity on the preparation of the data. First, we have found that independent measurements on Au-BPY-Au junctions using two completely different measurement methods (MCBJ/STM break junctions) yield very similar results (see Figure S1 in the SI), underpinning that the correlation plot and its principal components indeed act as specific fingerprints of the possible single-molecule junction evolution trajectories. Furthermore, the truncation of the data set, i.e., the removal of the trace-parts in the single-atom and in the low conductance tunneling regime, leaves the results of the PCP analysis for the two molecular configurations similar (see Figures S2 and S4 in the SI). On the other hand, the PCP-NN method is found to be especially sensitive to the temporal homogeneity of the data, inhomogeneous data portions may introduce severe artifacts to the analysis (see Figure S5 in the SI).

Generally, we can say, that the projections to a certain PC yield relevant classification criteria, if the character of the obtained two trace-classes (like their 1D or 2D histograms) fundamentally differ from each other as well as from the results of other applied PCP-s in the conductance region of our interest. As the PC-s with the highest eigenvalues represent the most important correlations in the data, the leading principal component projections usually fulfill these criteria. This is confirmed by our analysis on BPY (DAF) molecules, where the first 4 (2) PCs indeed yield 8 (4) highly relevant trace classes, including several junction evolution trajectories, that are imperceptible from the 1D and 2D histograms of the entire data set. Furthermore, as the PCP method relies on the internal correlations of the data set, even a completely featureless (flat) molecular histogram peak may hide fundamentally different correlation structures, which are uncovered by the PCP method. The anatomy of the latter is demonstrated in the SI (Figure S6) using three simple hypothetical trace classes, for which the correlation structures and the PCS can be analytically evaluated. Finally, we note that the projection to a certain PC yields only two trace classes, but one may also apply sequential PCPs;^52^ e.g., first the clear molecular traces are selected by the projection to the proper PC, and afterward a further PCP analysis is applied for this restricted data set, finding further subsets among the clear molecular traces.

*EPCP-Aided Interpretation of Single-Molecule 1/f-Type Noise Measurements*. As a next step we demonstrate that the PCP method may provide a clarified, configuration-specific insight into the measurement of further physical quantities. As mentioned in the introduction, the scaling of the 1/f-type noise amplitude with the junction conductance supplies fundamental information about the nature of the metal-molecule bonds.^11,12,14,18,21^ Here, we investigate the noise characteristics of the widely studied Au-BPY-Au single-molecule junctions. As an initial hypothesis, in the HighG configuration the aromatic ring also couples to the metallic apex which may introduce a significant through-space contribution to the transport, whereas the LowG configuration relies on the transport through the linkers, anticipating through-bond characteristics. The measured noise characteristics, i.e., the most probable relative conductance noise, Δ*G*/*G* is shown by black line in Figure 4a for the entire data set (see the light blue area graph in Figure 4c for the corresponding conductance histogram in the molecular region). More details on the noise analysis, including the preparation of the noise data, and the investigation of the 1/f-type slopes of the spectra are available in the SI (Figures S8 and S9). This curve indeed exhibits through-space-type Δ*G*/*G* ≈ const. characteristics at the top tail of the HighG peak, and through-bond-type Δ*G*/*G* ≈ *G*^–0.5^ characteristics at the bottom tail of the LowG peak. As a comparison, the orange dashed line represents the envisioned noise evolution according to the above-discussed considerations, i.e., through-space-type noise in the entire HighG region and through-bond-type noise in the entire LowG region. More precisely, the orange dashed line extrapolates the best fitting Δ*G*/*G* ≈ *G*^–0.5^/Δ*G*/*G* ≈ const. tendencies at the low/high conductance *sides* of the investigated conductance region to the entire conductance range assuming a sharp transition at the intersection of these lines. It is clear that in the intermediate conductance region the noise data clearly deviate from this supposed behavior. This intermediate conductance region, however, is positioned around the conductance boundary between the HighG and LowG configurations (*G* ≈ 4 × 10^–3^G_0_), where the largest diversity of the possible junction evolution paths appears according to the above PCP analysis. Therefore, the interpretation of the unexpected noise evolution would remain highly speculative as long as the noise contributions of the two molecular configurations are not separated very clearly.

**Figure 4:**
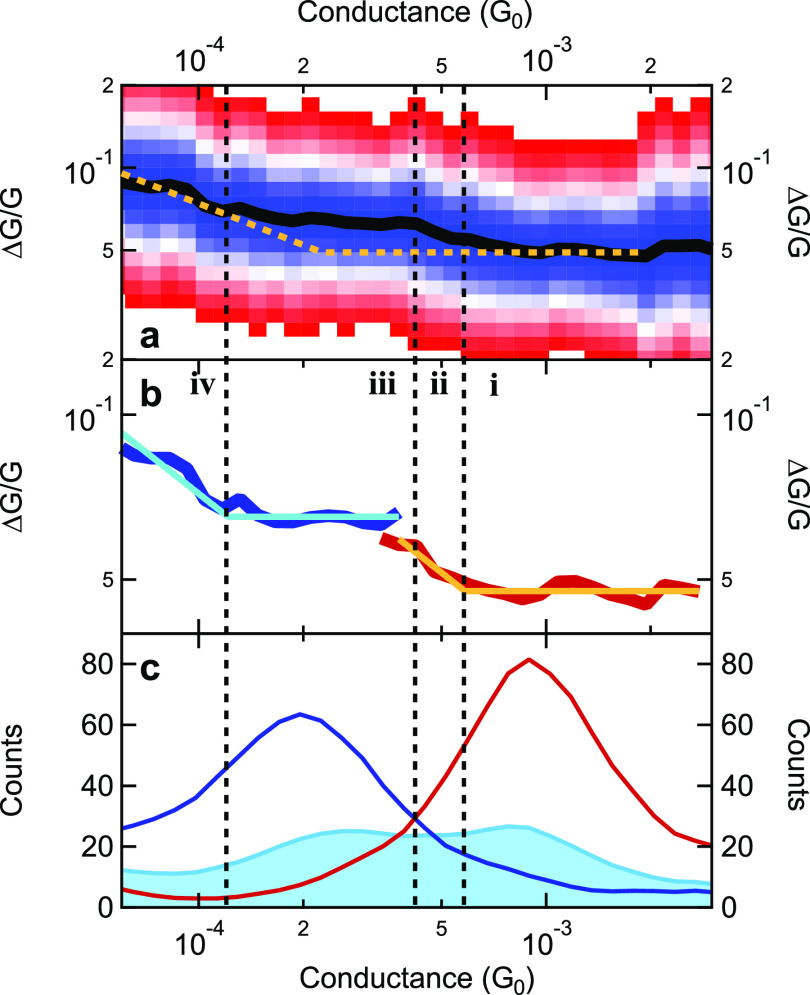
Configuration-specific 1/f-type noise analysis of Au-BPY-Au junctions. (a) Most probable relative conductance noise as a function of the conductance (black line) for the entire data set. The colorscale illustrates the scattering of the noise data (see SI for more details). The orange dashed line represents the hypothetical noise evolution discussed in the text. (b) Configuration-specific noise analysis demonstrating the noise evolution separately for the HighG (red) and LowG (blue) configurations. The orange and light blue lines represent the best-fitting model curves. (c) Conductance histogram of the entire data set (light blue area graph) together with the configuration-specific EPCP projections (blue and red lines). The roman numbers together with the black dashed boundary lines illustrate the four characteristic regions of the junction evolution, as discussed in the text.

The EPCP-based selection demonstrated by the red and blue histograms in Figure 1b offers a unique possibility to very clearly separate the HighG and LowG configurations, investigating their noise characteristics *separately*. The most probable Δ*G*/*G* values can be generated separately for these two data subsets, as demonstrated by the red and blue curves in Figure 4b. According to our hypothesis, we would expect Δ*G*/*G* ≈ const. for the HighG subset (red) and Δ*G*/*G* ≈ *G*^–0.5^ for the LowG subset (blue). This is, however, clearly contradicted by the data. Instead of the markedly *different* noise characteristics of the HighG and the LowG configurations, both configurations exhibit very *similar* noise evolution with through-space-type constant Δ*G*/*G* at the top part of the actual subset histogram peak, and through-bond-type characteristics (Δ*G*/*G* ≈ *G*^–0.5^) at the bottom tail of the subset histogram peaks. The latter tendencies are also illustrated in Figure 4b by the orange (light blue) line representing the best fitting model curves assuming *constant*/*G*^–0.5^ noise evolution above/below a certain conductance threshold for HighG (LowG) configurations. Again, the deviation from the expected behavior is the most striking in the intermediate conductance region, where both the HighG and the LowG data subsets exhibit *opposite* behavior to the initial hypothesis, i.e. through-bond-type behavior for the HighG configuration and through-space-type behavior for the LowG configuration.

According to this configuration-specific analysis, we can identify four distinct conductance intervals according to distinct noise evolution characteristics (see the roman numbers in panel Figure 4b and the separating dashed lines). (i) At the top part of the HighG peak the HighG configuration indeed exhibits additional π-coupling between the pyridine ring and the electrodes, giving rise to constant through-space-like noise characteristics. (ii) As the HighG configuration is stretched (i.e., at the bottom tail of the HighG peak) this coupling vanishes giving rise to increasing, through-bond-type noise characteristics. (iii) At the top part of the LowG peak again a significant constant, through-space noise contribution is observed, indicating that the pyridine ring couples to the side of the electrode in this region as well. (iv) Finally, as the LowG configuration is stretched, this π-coupling vanishes again, the molecule straightens out, and again increasing, through-bond-type noise characteristics are observed (bottom tail of the LowG peak). More precisely, the borders between the i/ii and iii/iv intervals are identified by the points, where the fitted noise characteristics (orange and light blue lines in panel (b)) turn from constant to Δ*G*/*G* ≈ *G*^–0.5^ dependency (*G* = 5.8 × 10^–4^G_0_ for i/ii and *G* = 1.2 · 10^–4^G_0_ for ii/iv), whereas the border between the ii/iii intervals is the crossing point of the HighG and LowG histograms in panel (c), *G* = 4.2 × 10^–4^G_0_. Note, however, that these conductance regions rely on a rather simplifed model, considering exactly ∼*G*^–0.5^ and ∼*G*^0^ scaling exponents with a sharp transition between these. It would be more realistic to permit deviations from these limiting scaling factors, and a more gradual transition between the two distinct behaviors, but due to the limited resolution of the noise data we rather apply the most simple model grabbing the most important rough tendencies.

The above, peak-specific insight into the noise characteristics explores the cases where solely the HighG or solely the LowG configurations appear. However, the noise evolution of the entire data set (black curve in Figure 4a), where the two molecular configurations often appear sequentially on the same trace, follows the same trends as the separated configurations (through-space/through-bond/through-space/through-bond characteristics in regions i/ii/iii/iv, respectively). This suggests that the above-described physical mechanisms are not specific to the separate, or the sequential appearance of the two configurations, but rather a specific conductance interval (i–iv) always shows similar noise evolution. In the case of the sequential appearance of the two configurations, we argue that around the ii/iii boundary, the molecule may jump to another bonding site, such that the pyridine ring again flips to the side of the electrode giving rise to a significant constant, through-space noise contribution. Note that the recurring presence of through-bond-type segments in the noise evolution also excludes the appearance of a dimer configuration (like the LowG configuration in the case of DAF molecules^11^), and as in the latter case the dimer junction should exhibit purely through-space-type noise characteristics.

In conclusion, we have demonstrated the merits of PCP-based unsupervised data classification in single-molecule break junction measurements. According to our analysis on room-temperature Au-BPY-Au and Au-DAF-Au single-molecule structures, all the leading principal components project the conductance traces to highly relevant data subsets, signaling alternative junction evolution trajectories which are obscured on the 1D and 2D conductance histograms. These results demonstrate an efficient classification according to the well-established and well-visualized internal correlations of the data set; i.e., the PCP-method lacks the disadvantage of the black-box-like nature of more complex machine learning approaches, as well as the subjective nature of custom-created manual data filtering algorithms.

Finally, we demonstrated that these efficient data classification capabilities can be exploited in the measurement of further physical quantities, where the mixture of alternative junction evolution trajectories would obscure the analysis. In particular, the PCP method enabled us to perform a configuration-specific 1/f-type noise analysis, uncovering refined details of the junction evolution: we identified the coupling of the aromatic ring to the electrodes through the π orbitals in two distinct conductance regions and its subsequent uncoupling as these configurations are stretched.

## Supporting Information Available

Demonstration of the sensitivity of the PCP analysis on the data preparation, tutorial illustration of the anatomy of the PCP method and the details of the noise analysis (PDF)
