# Supplementary material for: Configuration-Specific Insight into Single-Molecule Conductance and Noise Data Revealed by the Principal Component Projection Method
Source: J Phys Chem Lett. 2023 May 30;14(22):5109–18. doi: 10.1021/acs.jpclett.3c00677 (PMC10258846; doi:10.1021/acs.jpclett.3c00677)
Supplement: Supplementary file 1 — jz3c00677_si_001.pdf [file jz3c00677_si_001.pdf]

Supporting Information
Configuration-Specific Insight to Single-Molecule Conductance and Noise Data Revealed by Principal Component Projection Method

Z. Balogh,[†,‡] G. Mezei,[†,‡] N. Tenk,[†] A. Magyarkuti,[†] and A. Halbritter[*,†,‡]

†*Department of Physics, Institute of Physics, Budapest University of Technology and Economics, Műegyetem rkp. 3., H-1111 Budapest, Hungary.*

‡*ELKH-BME Condensed Matter Research Group, Műegyetem rkp. 3., H-1111 Budapest, Hungary.*

E-mail: halbritter.andras@ttk.bme.hu

Sensitivity of the PCP analysis on the data preparation

Comparison of datasets acquired with different experimental methods

To test the robustness of the PCP analysis, we compare the correlation matrices and the principal components for Au-BPY-Au junctions measured in two independent experiments, using two completely different measurement methods (MCBJ/STM break junctions), see Figure S1. The correlation matrix of the MCBJ dataset (Fig. S1a) indeed resembles that of the STM dataset (Fig. 1c in the manuscript) including fine details of the distinct positive/negative correlation lobes. Accordingly, the principal components are also very similar, as illustrated for the first two principal components in Fig. S1b,c (see the light/dark green lines for the MCBJ/STM data). This sample-to-

sample and method-to-method reproducibility (also confirmed by further measurements on other independent datasets) underpins that the correlation plot and its principal components indeed act as specific fingerprints of the possible single-molecule junction evolution trajectories.

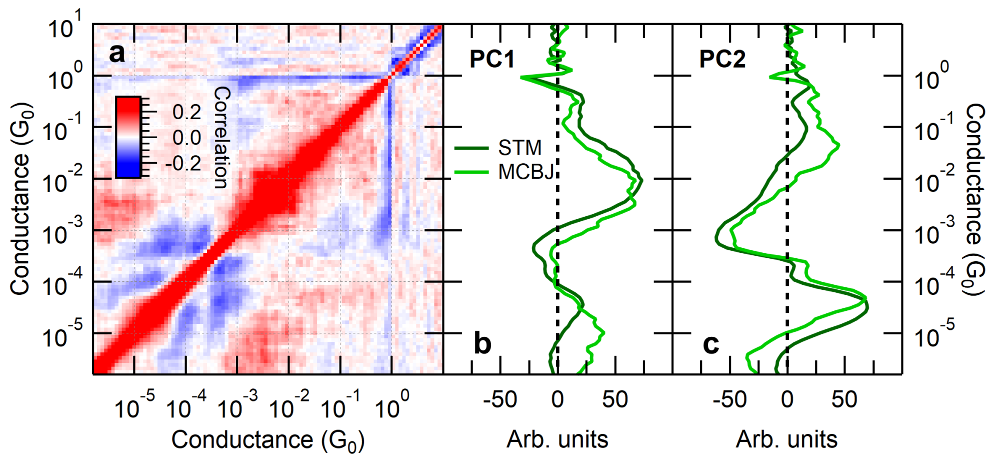

Figure S1: *The figure compares the correlation plot of an Au-BPY-Au MCBJ dataset (a) and the corresponding first two principal components (b,c) to STM break junction measurements, demonstrating excellent sample-to-sample and method-to-method reproducibility.*

Comparison of the PCs for full and truncated datasets

In the manuscript, we have shown that $P^{(1)}$ highlights a strong correlation between the single-atom ($G \approx 1\,\mathrm{G_0}$) configuration and the two molecular configurations of Au-BPY-Au single-molecule junctions. Next, we investigate the influence of this correlation on the overall structure of the principal components. In Fig. S2, the correlation matrix and the first four principal components of the Au-BPY-Au STM break junction measurements are demonstrated for a truncated dataset, where datapoints above $G = 0.67\,\mathrm{G_0}$ (single or few atom configurations) are excluded from the data. This threshold is set to the conductance value, where the correlations demonstrated by $P^{(1)}$ first vanish (cross zero) below $G = 1\,\mathrm{G_0}$. The such-obtained principal components (green lines in Figs. S2b,c,d,e) show highly similar patterns to the PCs of the full, untruncated dataset (black lines in Figs. S2b,c,d,e), i.e. the correlations in the molecular conductance region are not sensitive to the inclusion of the single-atom configurations in the dataset. On the other hand, it is emphasized

that in $P^{(1)}$, the correlations between the single-atom and single-molecule configurations are rather strong (see the magnified, linear-scale histograms of the corresponding PCPs in Fig. S3), i.e. it is still useful to include the single-atom configurations in the analysis as these configurations act as strong precursors of the subsequent molecular configurations.

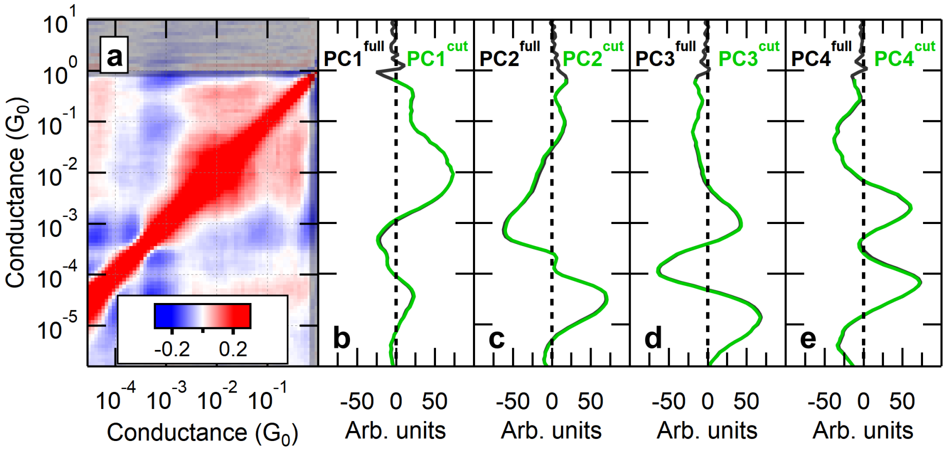

Figure S2: *Correlation matrix (a) and the first four principal components (b,c,d,e) for a truncated Au-BPY-Au dataset, where the datapoints with* $G > 0.67\,G_0$ *conductance are excluded from the analysis. The green lines show the PCs of the truncated dataset, whereas the black lines demonstrate the PCs of the original full dataset (the same as in Fig. 2 of the manuscript).*

As a next step, we check the effect of the data truncation: if not only the single or few atom configurations, but also the tunneling configurations below the HighG and LowG molecular conductance region ($G < 4.2 \cdot 10^{-5}\,G_0$) are excluded from the data. The such-obtained principal components (green lines in Figs. S4b,c,d,e) also show similar patterns to the PCs of the full, untruncated dataset (black lines in Figs. S4b,c,d,e), but two clear differences can be already noticed: (i) $P^{(3)}$ and $P^{(4)}$ are interchanged (i.e $P^{(3)}$ of the full dataset corresponds to $P^{(4)}$ of the truncated dataset and vice versa), and (ii) the low-conductance ends of the PCs (especially $P^{(3)}$ and $P^{(4)}$) are somewhat deformed. The latter is attributed to the orthogonality condition of the PCs. Note, that this deep tunneling region gives much stronger weights to the PCs than the single-atom region, and accordingly, the exclusion of the deep tunneling region necessarily yields the deformation of the PCs due to their orthogonality requirement. According to this analysis, it is recommended to choose a wide enough conductance region around the molecular conductance range of interest for the principal

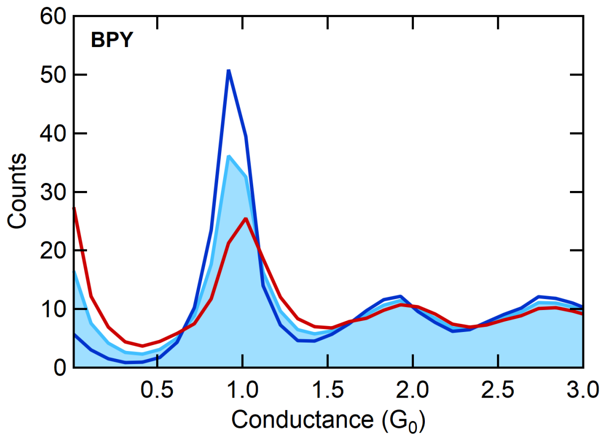

Figure S3: *Precursor features of the molecular configurations in the single-atom conductance region for Au-BPY-Au junctions. The Figure reproduces the single-atom conductance region of Fig. 2c1 in the manuscript using a magnified linear conductance scale. The light blue area graph represents the 1D histogram of the entire dataset, whereas the blue/red lines show the histograms for trace-class (a)/(b) obtained by PCP-NN method using $P^{(1)}$. Note, that a rather strong precursor effect is concerned, the contrast (i.e. the peak-height difference divided by the average peak-height at $1\,G_0$) exceeds 65%.*

component analysis.

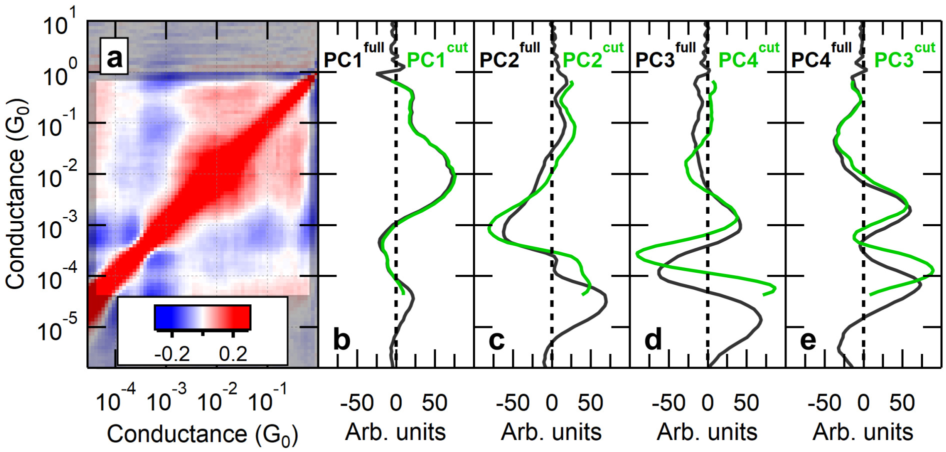

Figure S4: *Correlation matrix (a) and the first four principal components (b,c,d,e) for a truncated Au-BPY-Au dataset, where the datapoints with $G > 0.67\,G_0$ and $G < 4.2 \cdot 10^{-5}\,G_0$ conductance are excluded from the analysis. The green lines show the PCs of the truncated dataset, whereas the black lines demonstrate the PCs of the original full dataset (the same as in Fig. 2 of the manuscript).*

Finally, we demonstrate an aspect, where the PCP method is extremely sensitive to the data preparation. Fig. S5c shows the so-called temporal conductance histogram,[1] where the horizontal

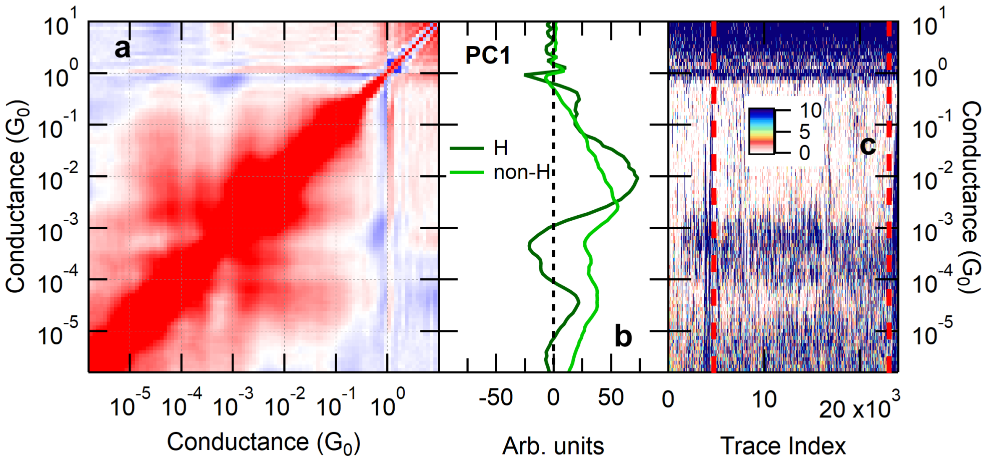

Figure S5: *The figure illustrates that the PCP analysis is extremely sensitive to the homogeneity of the investigated traces. Panel (c) shows the temporal histogram of the entire Au-BPY-Au STM dataset. If the inhomogeneous parts outside the interval bordered by red dashed lines are included in the analysis, both the correlation plot (a) and the principal components strongly deviate from the well-established behavior of the Au-BPY-Au system. The latter is illustrated in panel (b) comparing* $P^{(1)}$ *for homogeneous (dark green) and inhomogeneous (light green) datasets. Similar problems arise, if the bottom conductance cutoff is not set properly, and therefore the base noise level of the current amplifier is also included in the conductance region of the analysis (not shown).*

axis is the trace index, r, and the vertical cuts represent the single-trace conductance histograms, $N_i(r)$ as a colorscale. This plot visualizes the temporal evolution of the conductance traces. For a reliable measurement, the temporal homogeneity of the temporal conductance histogram is required. This is satisfied in the region between the red dashed lines, for which region the correlation matrix was calculated in Fig. 1c in the manuscript. Before the first dashed line, however, a region is observed, where the molecular structures are missing in the temporal histogram. Such an evident inhomogeneity in the data yields a completely different correlation plot (Figure S5a) than the correlation plot for the homogeneous portion (Fig. 1c in the manuscript), which also affects the principal components. Note, that Figure S5b only compares $P^{(1)}$ for the homogeneous and the non-homogeneous datasets, but all the leading principal components strongly deviate from the well-defined PCs of the homogeneous dataset. This comparison highlights a weak point of the PCP-NN analysis: special care must be taken to ensure the homogeneity of the conductance traces (and to check the results of the analysis for various independent homogeneous datasets), otherwise the results will reflect artifacts due to the data inhomogeneity instead of the well-defined internal

correlations.

Anatomy of the PCP method

To demonstrate the anatomy of the correlation structures, principal components and principal component projections, we perform a simple case study with hypothetical conductance traces. We consider a conductance region with N conductance bins, from which molecular features can be observed within M conductance bins in the conductance interval $G_{\text{min}} < G_{\text{molecular}} < G_{\text{max}}$. We consider different types of conductance traces: (i) a flat molecular plateau at a uniformly distributed random conductance within the molecular conductance region, and vertical conductance jumps before and after the molecular plateau (Fig. S6a1); (ii) A tilted molecular plateau spanning the entire molecular interval with vertical jumps before and after the plateau (light green line in Fig. S6a2); and (iii) a tunneling trace with a constant slope in the entire conductance region (brown line in Fig. S6a2). In the following we demonstrate the conductance histograms (b1,b2,b3), the correlation plots (c1,c2,c3), the key principal components (d1,d2,d3), and the principal component projection histograms (e1,e2,e3) for different mixtures of the above three types of traces (see panels a1,a2,a3). For these example traces all these plots can be analytically calculated. Note, that the conductance histograms (b1,b2,b3) are very similar for all the three mixtures (a flat molecular peak in the entire molecular region), but the correlation structures, PCs, and PCPs behind these flat histograms are completely different.

In the first case only type (i) traces are considered. In this case, the molecular region exhibits negative correlation with itself (blue rectangle in c1), the diagonal of the correlation plot is by definition unity, and the remaining parts of the correlation plot are uncorrelated (white parts). For this correlation plot, two important types of principal components are identified: the red curve (d1) represents a single (nondegenerate) eigenvector with $\lambda = 0$ eigenvalue, whereas $M-1$ linearly independent degenerate eigenvectors with $\lambda = M/(1-M)$ eigenvalue can be composed as the linear combinations of the blue type of eigenvectors (d1), i.e. $+1$ and -1 values at two different

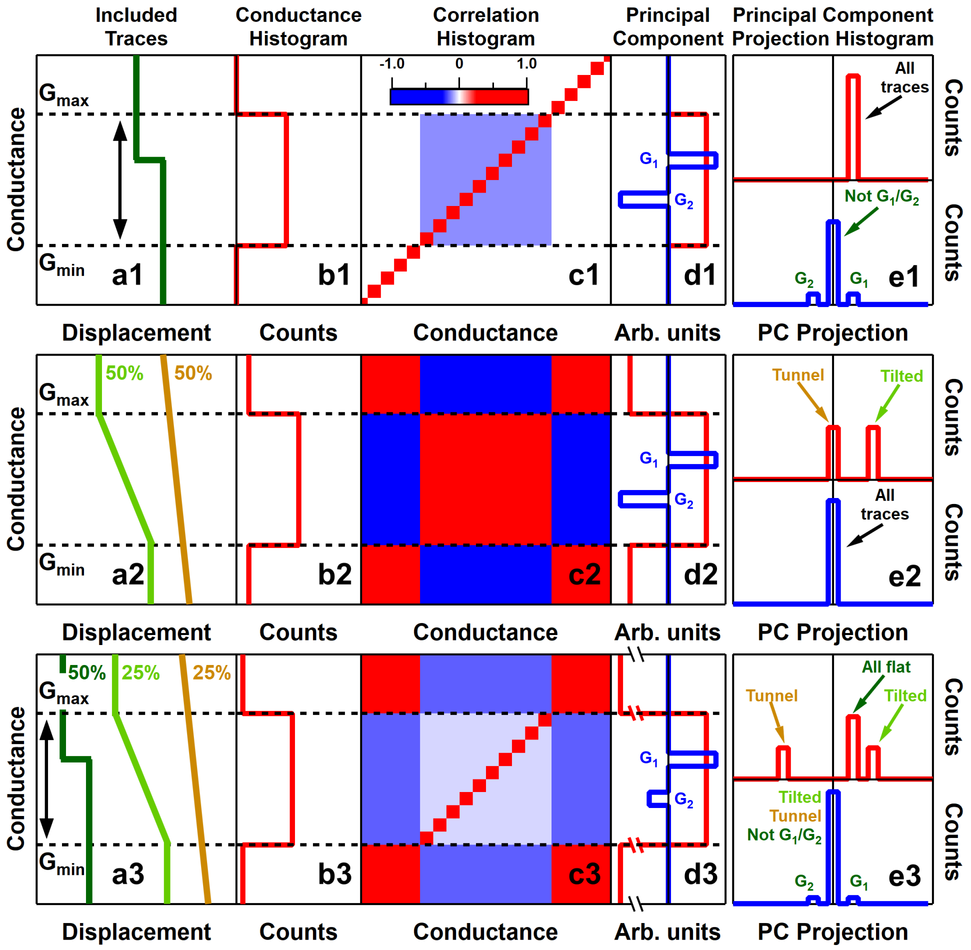

Figure S6: *Conductance histograms (b1,b2,b3), correlation plots (c1,c2,c3), key principal components (d1,d2,d3) and principal component projection histograms (e1,e2,e3) for three different mixtures of hypothetical traces (a1,a2,a3) with flat molecular plateaus (dark green), tilted molecular plateaus (light green) and tunneling character (light brown). The conductance histograms represent a completely flat molecular peak for all the cases, but the underlying correlation structures are completely different according to the chosen mixture. The key principal components represent similar structures for all mixtures: flat regions with different values inside/outside the molecular conductance range (red PCs) or positive/negative peaks at chosen G_1 and G_2 conductances (blue PCs). The labels in the PC projection histograms respectively show the PCP values obtained by the projection of a certain trace class to the red/blue PCs (top/bottom panels). Note, that the red/blue PCs yield non-selective projections for the first (a1)/second (a2) trace group. It is also noted, that the blue PCs are $M-1$ times degenerate, and a possible linearly independent set of blue PCs can be chosen as a negative peak at a fixed G_2 conductance for all the $M-1$ PCs, and positive peak at $M-1$ possible $G_1 \neq G_2$ values. The linear combination of this set may yield PCs being selective for arbitrary molecular conductances (negative/positive values at two different conductance intervals, for which a selection is desired).*

conductance bins (G_1 and G_2) within the molecular region, and zeros in the rest of the bins. The remaining $N-M$ eigenvectors exhibit zero values inside the molecular region. The latter PCs are not relevant for the molecular region and are not discussed. Note, that the red PC with zero eigenvalue yields *the same principal component projection for all the traces* (red PCP histogram in e1), i.e. this PC is useless for the classification of the traces. In contrast, the blue PC gives positive (negative) PCP if the conductance plateau is positioned at G_1 (G_2), and zero PCP is obtained for the rest of the traces, i.e. this PCP can be well applied to select traces with dedicated conductances.

Panels a2,b2,c2,d2,e2 correspond to the $50\%-50\%$ mixture of tilted molecular traces and tunneling traces. This case yields a completely different correlation plot (c2) with $+1$ (red) and -1 (blue) correlations. For this correlation histogram the relevant PCs (d2) are very similar to the previous case, but the role of the two types of principal components are interchanged. In this case, the red PC exhibits finite eigenvalue ($\lambda = N$), and well-separated PCPs for the tilted molecular and tunneling traces (red PCP histogram in e2), i.e. this red PC is perfect for the selection of tunneling vs. molecular traces, but it was useless in the previous example, where such traces were not available in the dataset. On the other hand, here the blue-type PCs yield zero eigenvalues with a degeneracy of $M-1$, giving the same PCP for all traces (blue curve in e2). These PCs would be selective for plat plateaus at dedicated conductances, but are useless here, where such traces are not available.

If the three types of traces are mixed (50% flat molecular trace, 25% tilted molecular trace, 25% tunneling trace, see a3) the correlation structures of (c1) and (c2) are mixed, yielding a more complex correlation plot (c3). In this case, the key types of PCS (blue and red PC in d3) are both relevant, providing different classification criteria. The red PC yields positive (negative) PCP for the two types of molecular traces (tunneling traces), whereas the blue PC with positive (negative) value at G_1 (G_2) yields positive PCP for flat plateaus at G_1, negative PCP for flat plateaus at G_2 and zero PCP for the rest of the traces.

The presented trace-classes somewhat resemble the PCPs in the first and third row of Fig. 2 in the manuscript. In the first row, the PCP classifies clear molecular traces spanning the entire

molecular conductance range (HighG+LowG) against more featureless conductance decays. These trace classes are represented by the simplified situation of tilted molecular plateaus in the entire molecular region and tunneling traces. In contrast, the third row classifies HighG versus LowG traces, i.e. it can select traces, where the plateaus are confined to much narrower conductance regions than the entire molecular conductance range. This case is represented by the extreme situation of completely flat plateaus in the above analysis. For BPY molecules, the double-peaked histogram cannot tell us, whether all molecular traces show both HighG and LowG plateaus or the dataset is a mixture of traces with pure HighG and pure LowG configurations, or HighG+LowG, pure HighG, pure LowG, and featureless traces are all available. The PCPs, however, clarify these features: if a certain trace class is available (not available), then the related PC will exhibit high (low) eigenvalue with strong (poor) trace selectivity. All this is even more striking in the above example, where absolutely no features are resolved by the wide and flat molecular histogram peak, but the PCPs are able to sort out the underlying fundamentally different trace classes.

In addition to the non-selective PCs with small eigenvalues it is also possible, that a certain PC has sufficiently large eigenvalue, but it is not selective *in the conductance range of our interest*, or in this conductance range it does not provide further, independent selection criteria compared to the other investigated PCs (e.g. the PCs with larger eigenvalues). The latter case is illustrated with the example of Au-DAF-Au junctions, where $P^{(3)}$ yields very similar selection in the conductance range of the molecular peaks as $P^{(2)}$. This is demonstrated in Fig. S7, where the dark green line in panel (d) shows $P^{(3)}$, and the related PCP-NN-based two datasets are shown by the two 2D histograms in panels (a) and (b), and by the red and blue 1D histograms in panel (c). In the molecular conductance range the selection by $P^{(3)}$ either highlights the one or the other molecular peak, similarly to $P^{(2)}$ (see Fig. 3c2 in the manuscript). Here, it should be also emphasized, that if only some types of traces are available in the dataset (like the tilted molecular and the tunneling traces in Fig. S6a2), then a certain PC either selects from these trace classes, or it is non-selective, i.e. it is obvious, that usually only a few leading principal components can yield relevant and independent selections with high contrast.

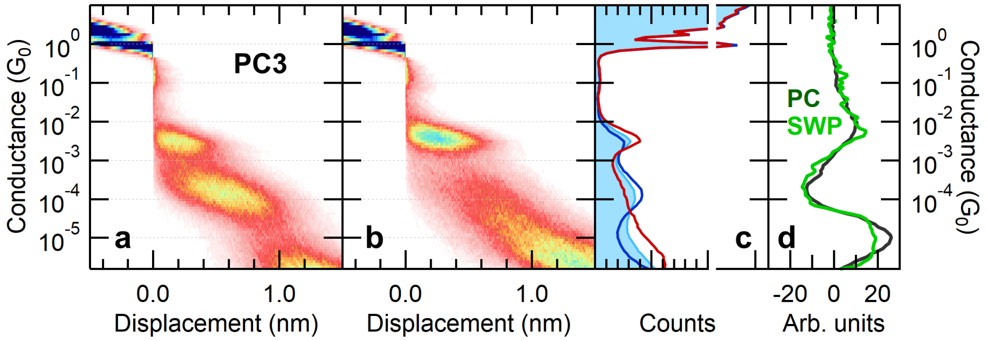

Figure S7: *PCP-NN analysis of Au-DAF-Au single-molecule junctions according to* $P^{(3)}$. *The corresponding principal component/SWP are shown by dark/light green lines in panels (d). Panels (a) and (b) respectively demonstrate the 2D histograms of trace classes (a) and (b). The corresponding 1D histograms are respectively shown by blue and red lines in panel (c) in comparison to the histogram of the entire dataset (light blue area graph). For this principal component the eigenvalue normalized to the eigenvalue of the first PC is* $\lambda^{(3)}/\lambda^{(1)} = 0.727$.

The basics of the noise measurements

The $1/f$-type noise analysis was carried out on conductance-displacement traces measured via the MCBJ method, using a special measurement system optimized for noise measurements. This includes a 24-bit resolution acquisition board with an anti-aliasing filter (NI-9239) and a low noise current amplifier (FEMTO DLPCA-200). Fig. S8a illustrates a single-molecule plateau with dominant HighG character on a conductance trace using a sampling rate of 50 kHz. Such conductance plateaus typically contain several thousands of data points. The noise spectra (light and dark blue lines in panel (b)) are calculated by fast Fourier transformation (FFT) for various, 256 datapoints long segments of the plateaus (see the regions denoted by light and dark blue arrows as examples in panel (a)), excluding the unstable trace-segments (i.e. where the conductance ratio between the beginning and the end of the evaluated trace segment is larger than a factor of two). Afterward, the noise spectrum is integrated for the $f = 2-5$ kHz frequency band yielding the mean squared conductance variation, $\langle(\Delta G)^2\rangle$ in this band. From this the $\Delta G/G = \sqrt{\langle(\Delta G)^2\rangle}/\langle G\rangle$ relative conductance noise is calculated at various mean conductances $\langle G\rangle$. In a certain conductance bin $\Delta G/G$ exhibits a broad scattering, and therefore it is not enough to use a single statistical mea-

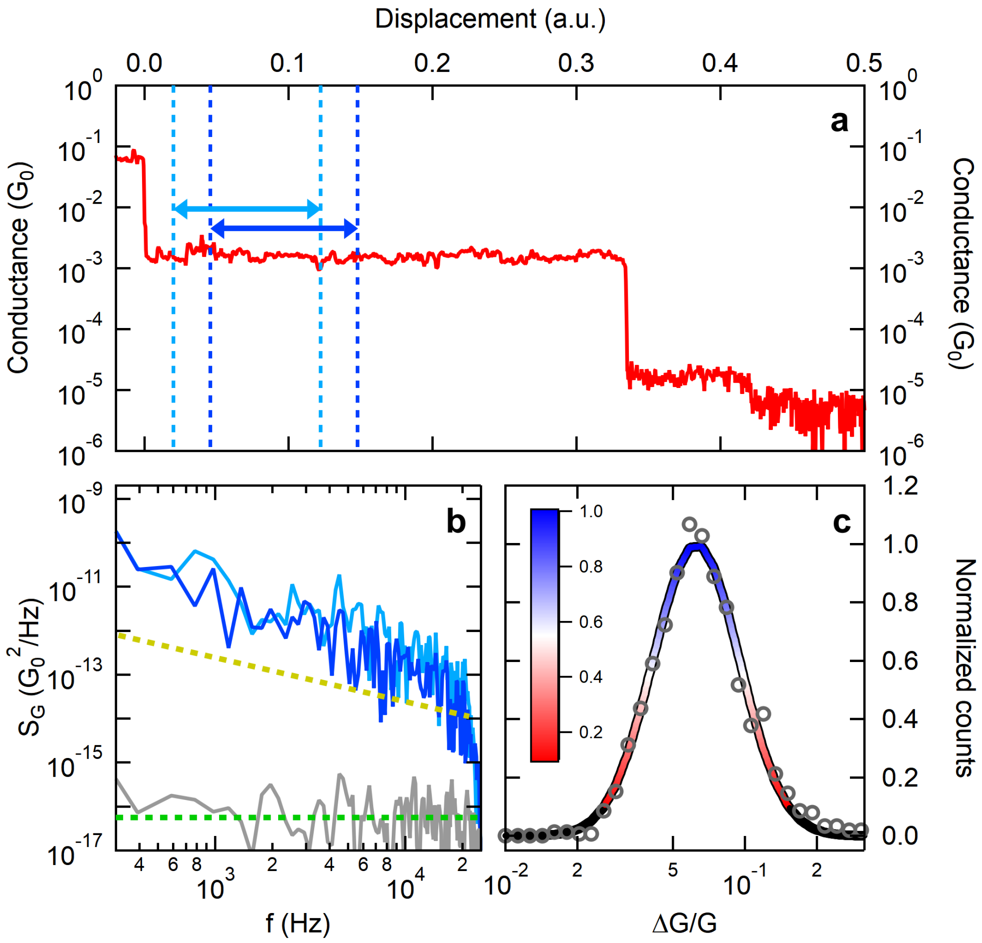

Figure S8: The noise spectra measured at 100 mV bias (light and dark blue lines in panel (b)) are calculated by FFT from fixed-length moving regions of the conductance traces (light and dark blue regions in (a)). In panel (b) the gray curve shows the significantly smaller noise floor of our measurement system in comparison to the expected thermal noise floor (green dashed line). The yellow dashed line illustrates the $S_G \sim 1/f$ frequency dependence. From the spectra, the relative conductance noise is calculated (see text), and the such obtained $\Delta G/G$ values are gathered to a histogram (20 bins/decade) for all the trace portions exhibiting a certain conductance (panel (c)). Gaussian fitting along the logarithmic x-axis is applied to determine the most probable $\Delta G/G$ value (i.e. the peak position of the Gaussian).

sure for the noise values, but it is important to visualize their entire distribution. The histogram of the such-obtained $\Delta G/G$ values is evaluated for all the trace-segments, for which the $\langle G \rangle$ average conductance falls into the same conductance bin (see panel (c)). All these histograms exhibit a Gaussian profile along the logarithmic $\Delta G/G$ axis, from which the most probable $\Delta G/G$ value is identified by the peak-position of the fitted Gaussian function (see the color-scaled fitting curve in

panel (c)). This is demonstrated by the black line in Fig. 4a in the manuscript representing the dependence of the most probable relative conductance noise on the mean conductance for the entire Au-BPY-Au dataset, as well as by the blue and red lines Fig. 4b in the manuscript representing the configuration specific noise data. Note, that the color-scale of the Gaussian fit in Fig. S8c encodes the ratio of the actual value on the Gaussian compared to the peak value. This color-coding is reproduced in Fig. 4a in the manuscript demonstrating the scattering of the noise data compared to the most probable noise value.

Frequency dependence of the noise

Each acquired noise spectrum was fitted by an $S_G \sim f^{\gamma}$ function within the $1-10\,\mathrm{kHz}$ frequency interval. The such obtained γ values were sorted according to the average conductance of the junction, and in the various conductance bins the histogram of the γ values was fitted with a Gaussian function. Fig. S9a shows the result of this analysis for the entire noise measurement dataset. The black line represents the most probable γ values, whereas the coloring represents the Gaussian fitting function using the same color-coding as in Fig. S8c. The orange dashed line shows the $\gamma = -1.4$ value, which was obtained in prior single-molecule 1/f-type noise measurements.[2] Fig. S9b shows the most probable γ values separately for the HighG and LowG configurations. As a reference, Fig. S9c shows the molecular conductance histogram peaks for the entire dataset (light blue) as well as for the two EPCP selections (red and blue lines, the same as in Fig. 4c of the manuscript).

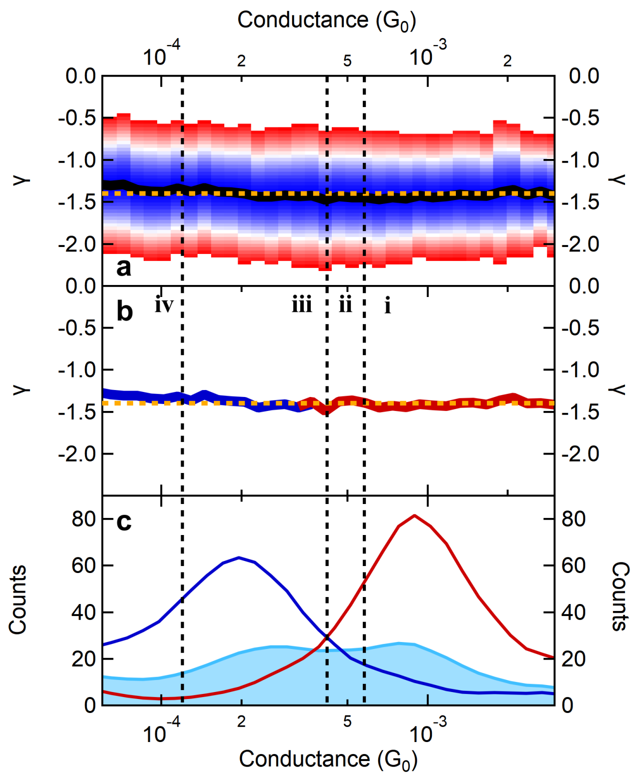

Figure S9: *(a) Most probable slopes (γ values) of the noise spectra obtained by fitting all noise spectra by the $S_G \sim f^\gamma$ function, and by fitting the histogram of the such-obtained γ values by a Gaussian for each conductance bin for the entire dataset. The colorscale illustrates the scattering of the noise data (see the text). The orange dashed line represents the $\gamma = -1.4$ value obtained in Ref. 2. (b) Configuration-specific analysis demonstrating the most probable γ values separately for the HighG (red) and LowG (blue) configurations. (c) Conductance histogram of the entire dataset (light blue area graph) together with the configuration-specific EPCP projections (blue and red lines). The roman numbers together with the black dashed boundary lines illustrate the four characteristic regions of the junction evolution, as discussed in the manuscript.*

References

(1) Magyarkuti, A.; Lauritzen, K. P.; Balogh, Z.; Nyáry, A.; Mészáros, G.; Makk, P.; Solomon, G. C.; Halbritter, A. Temporal Correlations and Structural Memory Effects in Break Junction Measurements. *J. Chem. Phys.* **2017**, *146*, 092319.

(2) Adak, O.; Rosenthal, E.; Meisner, J.; Andrade, E. F.; Pasupathy, A. N.; Nuckolls, C.; Hybertsen, M. S.; Venkataraman, L. Flicker Noise as a Probe of Electronic Interaction at Metal-Single Molecule Interfaces. *Nano Lett.* **2015**, *15*, 4143–4149.
